# Stepwise approach to curative surgery using percutaneous transhepatic cholangiodrainage and portal vein embolization for severe bile duct injury during laparoscopic cholecystectomy: a case report

**DOI:** 10.1186/s40792-016-0154-5

**Published:** 2016-03-17

**Authors:** Naruhiko Honmyo, Shintaro Kuroda, Tsuyoshi Kobayashi, Kohei Ishiyama, Kentaro Ide, Hiroyuki Tahara, Masahiro Ohira, Hideki Ohdan

**Affiliations:** Department of Gastroenterological and Transplant Surgery, Hiroshima University Hospital, Hiroshima University, 1-2-3 Kasumi, Minami-ku, Hiroshima, 734-8551 Japan

**Keywords:** Acute cholecystitis, Bile duct injury, Laparoscopic cholecystectomy, Portal vein embolization, Posthepatectomy liver failure, ^99m^Tc-galactosyl human serum albumin single-photon emission CT/CT fusion imaging

## Abstract

Laparoscopic cholecystectomy (LC) has been recently adapted to acute cholecystitis. Major bile duct injury during LC, especially Strasberg-Bismuth classification type E, can be a critical problem sometimes requiring hepatectomy. Safety and definitive treatment without further morbidities, such as posthepatectomy liver failure, is required. Here, we report a case of severe bile duct injury treated with a stepwise approach using ^99m^Tc-galactosyl human serum albumin (^99m^Tc-GSA) single-photon emission computed tomography (SPECT)/CT fusion imaging to accurately estimate liver function.

A 52-year-old woman diagnosed with acute cholecystitis underwent LC at another hospital and was transferred to our university hospital for persistent bile leakage on postoperative day 20. She had no jaundice or infection, although an intraperitoneal drainage tube discharged approximately 500 ml of bile per day. Recorded operation procedure showed removal of the gallbladder with a part of the common bile duct due to its misidentification, and each of the hepatic ducts and right hepatic artery was injured. Abdominal enhanced CT revealed obstructive jaundice of the left liver and arterial shunt through the hilar plate to the right liver. Magnetic resonance cholangiopancreatography revealed type E4 or more advanced bile duct injury according to the Bismuth-Strasberg classification. We planned a stepwise approach using percutaneous transhepatic cholangiodrainage (PTCD) and portal vein embolization (PVE) for secure right hemihepatectomy and biliary-jejunum reconstruction and employed ^99m^Tc-GSA SPECT/CT fusion imaging to estimate future remnant liver function. The left liver function rate had changed from 26.2 % on admission to 26.3 % after PTCD and 54.5 % after PVE, while the left liver volume rate was 33.8, 33.3, and 49.6 %, respectively. The increase of liver function was higher than that of volume (28.3 vs. 15.8 %). On postoperative day 63, the curative operation, right hemihepatectomy and biliary-jejunum reconstruction, was performed, and posthepatectomy liver failure could be avoided.

Careful consideration of treatment strategy for each case is necessary for severe bile duct injury with arterial injury requiring hepatectomy. The stepwise approach using PTCD and PVE could enable hemihepatectomy, and ^99m^Tc-GSA SPECT/CT fusion imaging was useful to estimate heterogeneous liver function.

## Background

Laparoscopic cholecystectomy (LC) is the standard treatment for symptomatic gallstones [1], and recently, early LC has been considered a feasible procedure for acute cholecystitis [2–4]. However, several studies have reported incidence rates of bile duct injury associated with LC ranging from 0.2 to 0.6 % [5–8], and major bile duct injury continues to be a clinical problem with significant morbidity for patients [9]. Bile duct injuries range from very minor accessory duct injuries to complicated hilar injuries, and Strasberg et al. categorized laparoscopic injuries to the biliary tract with a system known as the Strasberg-Bismuth classification [10]. Major bile duct injuries, such as Strasberg-Bismuth classification type E, tend to require biliary-enteric reconstructions, with a small proportion of patients possibly requiring hepatectomy [11] or even liver transplantation [12], and can extremely decrease patients’ quality of life. Therefore, prevention of bile duct injuries is a standard practice, and treatment strategies for major bile duct injuries should be considered carefully to minimize invasiveness but maintain curability without morbidities over the short and long terms.

If major hepatectomy is required, accurate estimation of the functional reserve of the future remnant liver is crucial to prevent posthepatectomy liver failure (PHLF) [13]. Conventionally, postoperative remnant liver function has been estimated based on remnant liver volume rate using computed tomography (CT) volumetry [13–15]. However, in some cases, function is not homogenous over the whole liver [16, 17], and thus, accurate estimation of regional liver function is important. Recently, some reports have indicated that ^99m^Tc-galactosyl human serum albumin (^99m^Tc-GSA) single-photon emission CT (SPECT)/CT fusion examination can accurately estimate regional liver function; ^99m^Tc-GSA scintigraphy reflects the number and function of hepatocytes, while CT provides precise anatomical information [18–21]. Here, we report a case of successful hepatectomy and biliary reconstruction after severe bile duct injury associated with LC, in which a stepwise approach was taken, using ^99m^Tc-GSA SPECT/CT fusion imaging to estimate the future remnant liver function.

## Case presentation

A 52-year-old woman with hypertension, who had undergone appendectomy at 23 years old and total hysterectomy for uterine myoma at 41 years old, visited a clinic with chief complaints of fever and upper abdominal pain. She had previously experienced gallstone attacks, and emergency LC was performed upon diagnosis of acute cholecystitis on the following day. During the operation after cholecystectomy, the surgeons found bile leakage and clipped the points of leakage with placement of an intraperitoneal drainage tube. As bile leakage persisted after the operation, she was transferred to our university hospital for radical cure on postoperative day 20.

On admission, she had no fever, jaundice, and abdominal pain. The abdominal cavity drainage tube discharged approximately 500 ml of bile juice in a day. In laboratory studies, although liver and bile duct enzymes slightly were increased, total bilirubin level was in the normal range (aspartate aminotransferase, 60 IU/l; alanine aminotransferase, 112 IU/l; alkaline phosphatase, 1855 IU/l; γ-glutamyl transpeptidase, 209 IU/l; and total bilirubin, 1.2 mg/dl). A slight inflammatory reaction was also detected, with white blood cell count of 8680/mm^3^ and C-reactive protein level of 3.6 mg/dl. Indocyanine green (ICG) retention rate after 15 min (ICG-R15) was 4.9 %, and ICG fractional disappearance rate (K-ICG) was 0.201.

**Fig. 1:**
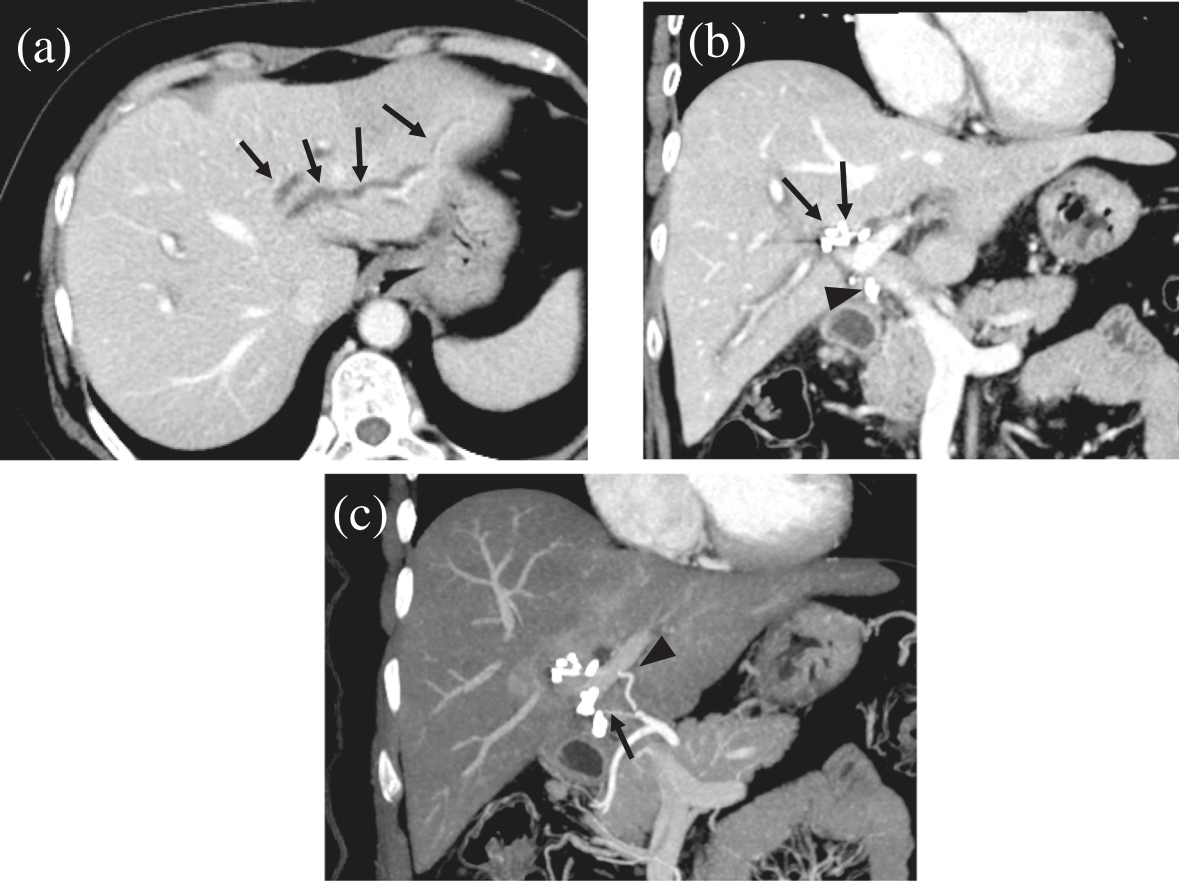
Abdominal enhanced computed tomography (CT) reveals visible expansion of the intrahepatic bile duct of the left lobe of the liver (**a** *arrow*). A part of the common bile duct is not shown because of resection during laparoscopic cholecystectomy, and some clips are present around the hilus hepatis region (*arrow*) and lower part of the common bile duct (*arrowhead*) (**b**). The common hepatic artery branches normally into the right hepatic artery (RHA) and left hepatic artery (LHA). The RHA is cut off and clipped (*arrow*), and a hilar shunt from the LHA (*arrowhead*) maintained feeding of the right liver via collateral circulation (**c**)

**Fig. 2:**
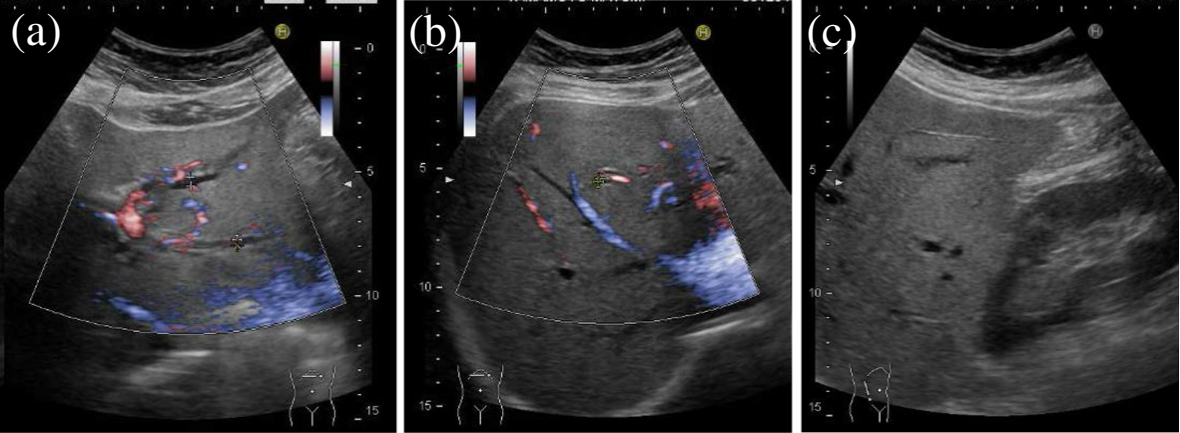
Abdominal ultrasound examination reveals the intrahepatic bile ducts in the left lobe expanded up to 3.3 mm (**a**) and slight enlargement of the intrahepatic duct in the right lobe to 1.7 mm (**b**). Hepato-renal contrast is observed, which suggested the possibility of fatty liver (**c**)

**Fig. 3:**
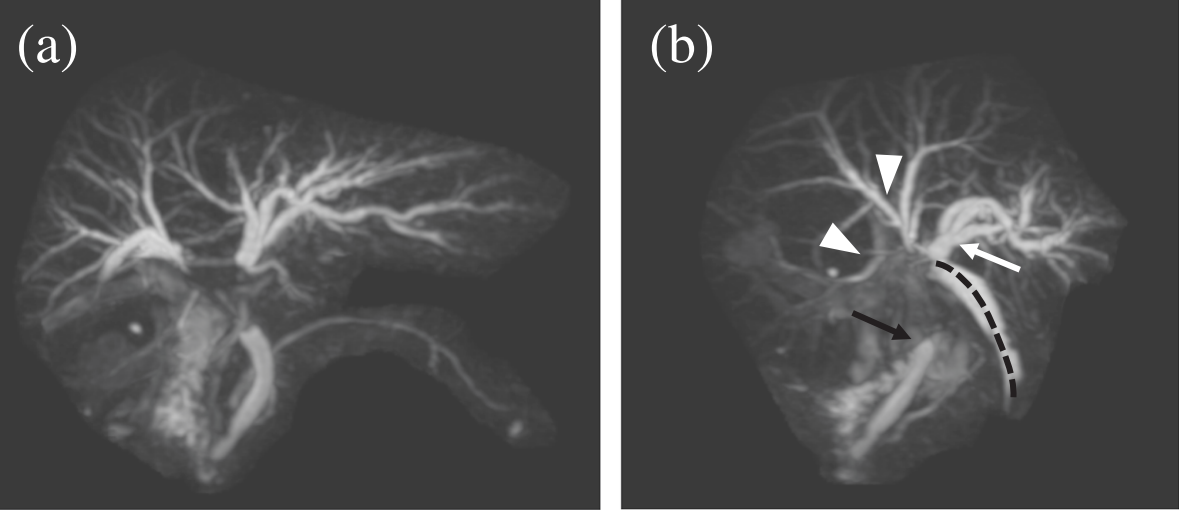
Postoperative magnetic resonance cholangiopancreatography (MRCP) reveals that the left hepatic duct and hepatic ducts of the right anterior and posterior sections are completely disconnected, which is classified as type E4 bile duct injury according to the Bismuth-Strasberg classification (**a**). Right-side view of the MRCP (**b**), the *white arrow* indicates the left hepatic duct, the *white arrowheads* indicate the hepatic ducts of the anterior and posterior sections, and the *black arrow* indicates the end of the remnant lower common bile duct. The *black dashed line* outlines the intraperitoneal drainage tube placed near the injured hilus hepatis

Abdominal enhanced CT revealed that the intrahepatic bile duct in the left liver was occluded and dilated by clips around the hilus hepatis region and the common bile duct was absent (Fig. 1a, b). Arterial feed to the right liver was maintained by shunting through the hilar plate due to scission of the right hepatic artery (RHA) during previous operation (Fig. 1c). Abdominal ultrasound examination revealed intrahepatic bile ducts expanded up to 3.3 mm in the left lobe, and hepato-renal contrast suggested the possibility of fatty liver (Fig. 2). Magnetic resonance cholangiopancreatography (MRCP) revealed type E4 bile duct injury according to the Bismuth-Strasberg classification [10]: the left hepatic duct, hepatic duct of the right anterior section, and that of the right posterior section were all disconnected (Fig. 3a, b).

**Fig. 4:**
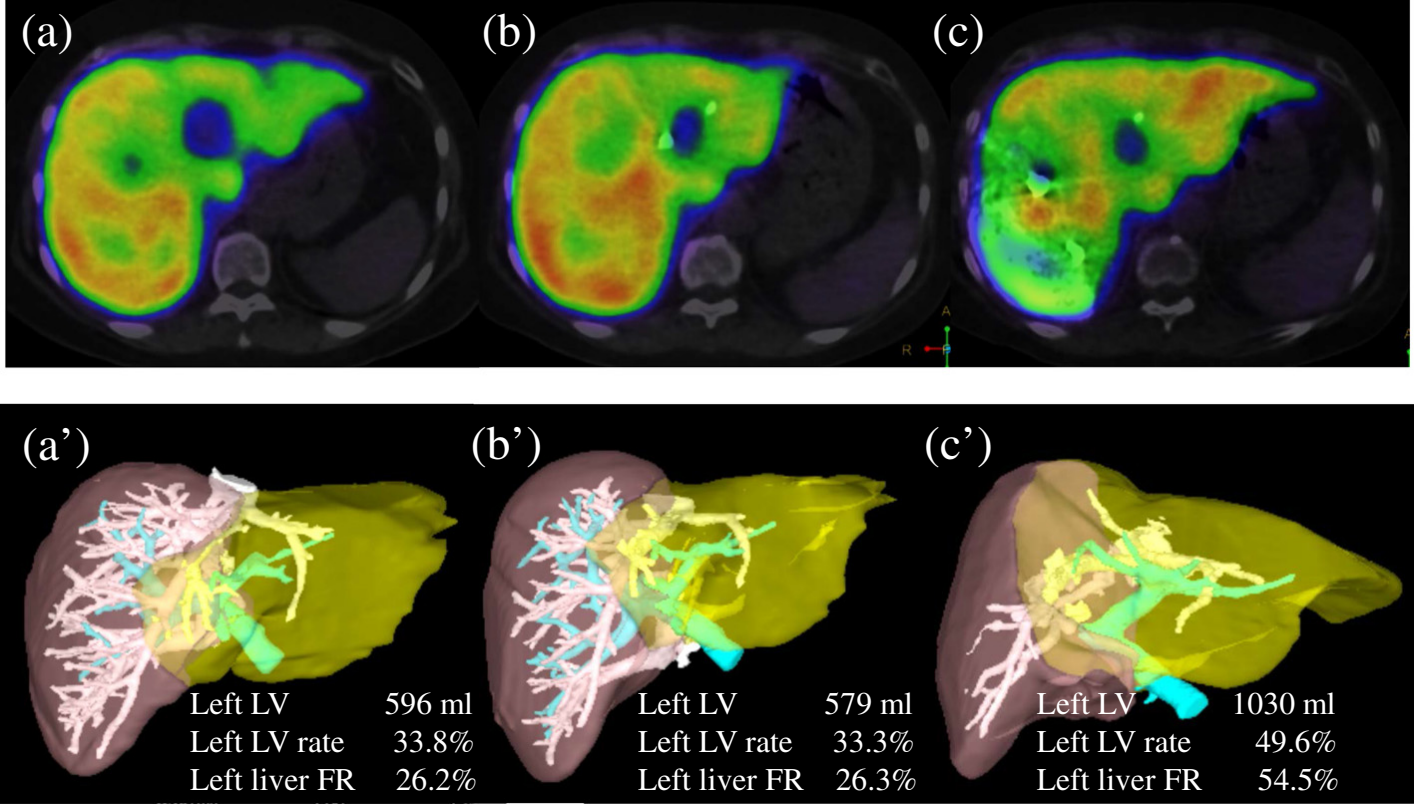
A sequential transition of liver function was shown visually. On admission, ^99m^Tc-galactosyl human serum albumin (^99m^Tc-GSA) single-photon emission computed tomography (SPECT) revealed the superior uptake of hepatocytes in the right liver compared with the left (**a**), and the volume of the right liver was also greater in quantity on CT volumetry: the left liver volume, the left liver volume rate of the whole liver, and the left liver function rate were 596 ml, 33.8 %, and 26.2 %, respectively (**a**’). Two weeks after percutaneous transhepatic cholangiodrainage, an extent of the uptake of hepatocytes in the left liver was almost unchanged on ^99m^Tc-GSA SPECT (**b**), and the volume of the left liver was also stable on CT volumetry: the left liver volume, the left liver volume rate, and the left liver function rate were 579 ml, 33.3 %, and 26.3 %, respectively (**b**’). After two more weeks following portal vein embolization, ^99m^Tc-GSA SPECT revealed the greatly improved uptake of hepatocytes in the left liver (**c**), and CT volumetry revealed the swelled left liver almost equal to the right liver: the left liver volume, the left liver volume rate, and the left liver function rate were 1030 ml, 49.6 %, and 54.5 %, espectively (**c**’). *LV* liver volume, *FR* function rate

Although the total liver volume measured by CT volumetry on admission to our hospital was 1764 ml, the right and left liver volumes were 1168 ml (66.2 %) and 596 ml (33.8 %), respectively. ^99m^Tc-GSA SPECT/CT fusion imaging revealed that the function rate of the left liver was decreased because of obstructive jaundice, with function rate of 73.8 % in the right liver and 26.2 % in the left liver (Fig. 4a, a’). The remnant liver K-ICG, calculated by multiplying K-ICG by CT volume rate, was 0.068.

From the above results, we planned right hemihepatectomy and biliary-jejunum reconstruction. As a first step, percutaneous transhepatic cholangiodrainage (PTCD) to the left liver was performed to recover left liver function, which declined because of bile congestion on postoperative day 22 (Table 1). Two weeks later, the volume of bile drained by PTCD, which reflected left liver function, increased to about 120 ml per day, and the volume drained by the intraperitoneal tube, which reflected right liver function, decreased from 500 ml to about 400 ml per day. However, left liver function estimated by ^99m^Tc-GSA SPECT/CT fusion imaging results were unchanged: the total liver volume was 1738 ml, right liver volume was 1159 ml (66.7 %) with estimated function rate of 73.7 %, and left liver volume was 579 ml (33.3 %) with estimated function rate of 26.3 % (Fig. 4b, b’). The remnant liver K-ICG was stable at 0.067 (Table 1).

**Table 1:** Sequential data for the left liver (future remnant liver)

	Pre-PTCD	Post-PTCD	Post-PVE
Drain bile volume (ml)	500.0	390.0	143.8
PTCD bile volume (ml)	–	117.5	250.0
Bile bilirubin level of PTCD (mg/dl)	–	21.9	71.6
Estimated remnant liver volume (ml)	596.3	578.6	1029.9
Estimated remnant liver volume rate (%)	33.8	33.3	49.6
Estimated remnant liver function rate (%)	26.2	26.3	54.5
Remnant liver K-ICG	0.068	0.067	0.100

Post-PTCD and post-PVE indicate 2 weeks after PTCD and PVE, respectively. Drain refers to intraperitoneal drain, and bile volume is the mean value calculated from 4 days around the procedures except for Pre-PTCD

*PTCD* percutaneous transhepatic cholangiodrainage, *PVE* portal vein embolization, *K-ICG* indocyanine green fractional disappearance rate

To improve left liver function, portal vein embolization (PVE) of the right liver was performed as a next step on postoperative day 37. More than 2 weeks after PVE, bile drained by PTCD increased to about 250 ml, and bile drained by the intraperitoneal tube decreased to about 140 ml per day. The function shifted from the right to the left liver, and thus, function of the left liver improved rapidly: the total liver volume was 2078 ml, right liver volume was 1048 ml (50.4 %), and left liver volume was 1030 ml (49.6 %). Right liver function rate decreased to 45.5 %, and left liver function rate increased to 54.5 % (Fig. 4c, c’). The remnant liver K-ICG increased to 0.100 (Table 1).

On postoperative day 63, the radical operation of right hemihepatectomy and biliary-jejunum reconstruction was performed. After the operation, maximum total bilirubin increased to 6.9 mg/dl on postoperative day 2, and hyperbilirubinemia persisted even on postoperative day 5, with total bilirubin of 2.3 mg/dl. Although bile leakage of the biliary-jejunum anastomosis occurred, it was curable by conservative therapy. The patient left our hospital on postoperative day 32 from the second operation. One year after the second operation, no long-term morbidities such as stricture of the anastomosis had occurred.

### Discussion

Bile duct injuries associated with LC occur in patients who have acute or chronic inflammation that obscures the normal planes of the cystohepatic triangle or when the common bile duct is anatomically misidentified [22, 23]. In particular, bile duct injuries of Strasberg-Bismuth classification type E caused by misidentifying the common bile duct as a cystic duct frequently accompany arterial injuries; therefore, hepatectomy may be required for curative treatment [11].

The present case was type E4 or greater advanced bile duct injury with RHA injury; the left hepatic duct and hepatic ducts of the right anterior and posterior sections were completely disconnected. As a radical procedure, we selected right hemihepatectomy and biliary-jejunum reconstruction. We considered the alternative of biliary-enteric reconstruction only, involving anastomosis of three disconnected hepatic ducts with the jejunum. However, since arterial feeding of the right liver was supplied by hilar shunt because of the absence of the RHA, postoperative morbidities, such as leakage or stricture of anastomosis, could be involved as a result of bile duct ischemia [24, 25]. Additionally, hepatic ducts of the right anterior and posterior sections injured at the intrahepatic level would make anastomosis technically impractical.

In cases of an injury to both the bile duct and the hepatic artery, immediate repair of the RHA has shown good results, but repair of the RHA is rarely possible and the overall benefit is unclear [26]. In this case, RHA reconstruction would not have been effective due to the sufficient time that had passed (3 weeks from the first operation), allowing for the growth of collateral arterial flow, difficulty of RHA repair, and risk of postoperative anticoagulation.

Right hemihepatectomy might be possible based on estimation by remnant liver K-ICG on admission. However, the remnant liver function rate estimated by ^99m^Tc-GSA SPECT/CT fusion imaging was 26.2 %, and fatty liver was suspected on ultrasound examination. Therefore, immediate hepatectomy was considered likely to cause PHLF. Instead, a stepwise approach was selected involving PTCD to the left liver and PVE to the right. Hyperbilirubinemia, with total bilirubin of 2.3 mg/dl, was observed at postoperative day 5, but serum international normalized ratio (INR) was within the normal limit at 1.15. The International Study Group of Liver Surgery defines PHLF as an increased INR and concomitant hyperbilirubinemia on or after postoperative day 5 [27]. In this case, PHLF grade B had occurred, which is manageable without invasive treatment. The peak serum bilirubin value, observed on postoperative day 2, was high at nearly 7 mg/dl. Kishi et al. reported that a peak serum bilirubin of 7 mg/dl was predictive of liver failure in patients with a small future liver remnant among 301 patients who underwent an extended right hepatectomy [28]. Therefore, the volume rate of 50.4 % and function rate of 45.5 % after PVE in this case approached the limit for right hemihepatectomy, and the decision for hepatectomy based on ICG-R15 and function rate could lead to successful treatment [29].

To accurately estimate liver function, some recent reports have indicated the utility of ^99m^Tc-GSA SPECT/CT fusion examination [18–21]. Additionally, ^99m^Tc-GSA SPECT/CT fusion imaging is also useful for cases associated with PVE [19]. In this case, we had estimated the remnant liver function using ^99m^Tc-GSA SPECT/CT fusion imaging at three times: on admission, after 2 weeks of PTCD, and after 2 more weeks following PVE. The liver function rate calculated by ^99m^Tc-GSA SPECT/CT fusion imaging was lower than the liver volume rate indicated by CT volumetry in the left liver, which had been damaged because of bile congestion (function vs. volume, 26.2 vs. 33.8 %). On the other hand, 2 weeks after PVE, the liver function rate was higher than the liver volume rate in the left liver (function vs. volume, 54.5 vs. 49.6 %). The increase of liver function was significantly higher than that of the volume (function vs. volume, 28.3 vs. 15.8 %). Beppu et al. suggested that the true post-PVE liver function is possibly underestimated by traditional CT volumetry [19]. Similarly, the variation of function in the liver that has been damaged rapidly but not yet atrophied may not parallel changes in liver volume, as in this case. The present case indicates that CT volumetry may reveal overestimation of the liver with rapid dysfunction and underestimation of the liver with compensatory swelling. Preoperative underestimation of future remnant liver function after PVE may not increase the risk of postoperative morbidity; however, overestimation can lead to life-threatening PHLF. To estimate the remnant liver function, ^99m^Tc-GSA SPECT/CT fusion imaging may be more accurate than K-ICG test, especially in case of heterogeneous liver function such as ours. Conventionally in Japan, major hepatectomy, such as right or extended right hemihepatectomy and trisectionectomy, is allowed in patients with normal liver function (total bilirubin and ICG-R15 are in the normal range) [13, 15]. Thus, we assessed that hepatectomy up to 60 % of liver function rate, as a cutoff value, can be justified adequately in this case in accordance with it. Accumulation of cases in which the liver function associated with PVE is estimated by this useful modality is required.

Our patient had fatty liver but not hepatic cirrhosis, so compensatory functional improvement after PVE was considered to be sufficient. However, once PVE has been performed, right hemihepatectomy is unavoidable because of reduced right liver function and a foreign body in the right portal vein; therefore, the decision to perform PVE should be made carefully and requires proof of no remaining intrahepatic bile duct expansion and bile production of the left liver after PTCD. The level of bilirubin per day in drained bile can be a useful parameter of liver function [30], and bilirubin levels over 20 mg/dl in concentrated bile drained from PTCD with maintained volume indicated that the left liver function was improving. These stepwise processes permitted the radical cure and safer treatment at an appropriate time.

## Conclusions

We report a case of severe bile duct injury associated with LC and treated by a stepwise approach with PTCD and PVE to reach critical remnant liver function, with frequent estimation of liver function using ^99m^Tc-GSA SPECT/CT fusion imaging. This prudent stepwise process made right hemihepatectomy possible, and ^99m^Tc-GSA SPECT/CT fusion imaging may be a useful modality for heterogeneous liver function.

## Consent

Written informed consent was obtained from the patient for publication of this Case report and any accompanying images. A copy of the written consent form is available for review by the Editor-in-Chief of this journal.

## Abbreviations

^99m^Tc-GSA: ^99m^Tc-galactosyl human serum albumin
CT: computed tomography
ICG: indocyanine green
ICG-R15: ICG retention rate after 15 min
INR: international normalized ratio
K-ICG: ICG fractional disappearance rate
LC: laparoscopic cholecystectomy
MRCP: magnetic resonance cholangiopancreatography
PHLF: posthepatectomy liver failure
PTCD: percutaneous transhepatic cholangiodrainage
PVE: portal vein embolization
RHA: right hepatic artery
SPECT: single-photon emission CT
